# Impact of CDK Inhibitors on *TBXT* Expression in Chordoma Cell Lines Including the First Stable Cell Line of a High-Grade Chordoma

**DOI:** 10.3390/diagnostics14101028

**Published:** 2024-05-16

**Authors:** Sarah Bette, Luisa Haase, Juliane Nell, Thomas Grieser, Alexandra von Baer, Markus Schultheiss, Ralf Marienfeld, Peter Möller, Thomas F. E. Barth, Kevin Mellert

**Affiliations:** 1 Institute of Pathology, University Hospital Ulm, 89081 Ulm, Germany; 2 Institute of Radiology, University Hospital Augsburg, 86156 Augsburg, Germany; 3 Department of Trauma Surgery, University Hospital Ulm, 89081 Ulm, Germany

**Keywords:** chordoma, brachyury, dinaciclib, THZ1, cell line

## Abstract

Chordomas are very rare malignant neoplasms of the bone occurring almost exclusively along the spine. As the tumours are thought to arise from notochordal remnants, the vast majority of chordomas express the *TBXT* gene, resulting in detectable nuclear amounts of its gene product brachyury. This T-Box transcription factor is commonly recognised as being essential in chordoma cells, and limiting *TBXT* expression is thought to be the key factor in controlling this tumour. Although the tumour is rare, distinct molecular differences and vulnerabilities have been described with regard to its location and the progression status of the disease, rendering it mandatory for novel cell lines to reflect all relevant chordoma subtypes. Here, we describe a novel chordoma cell line arising from the pleural effusion of a disseminated, poorly differentiated chordoma. This cell line, U-CH22, represents a highly aggressive terminal chordoma and, therefore, fills a relevant gap within the panel of available cell culture models for this orphan disease. CDK7 and CDK9 inhibition was lately identified as being effective in reducing viability in four chordoma cell lines, most likely due to a reduction in brachyury levels. In this study, we determined the capability of the CDK7 inhibitor THZ1 and the CDK1/2/5/9 inhibitor dinaciclib to reduce *TBXT* expression at mRNA and protein levels in a broad range of nine cell lines that are models of primary, recurrent, and metastasised chordoma of the clivus and the sacrum.

## 1. Introduction

Chordomas are rare malignant bone tumours of notochordal origin with an incidence of 0.8 cases/1,000,000 p.a. and a slight predominance in men (1.8:1) [1]. Chordomas are typically diagnosed in elderly patients between 50 and 70 years of age. Paediatric chordoma patients (defined as patients up to 20 years of age) are a rare subgroup accounting for less than 5% of the patients [2,3]. Chordomas arise almost exclusively along the spine. They are often localised at the sacrum, representing roughly 50% of all chordomas, followed by chordomas of the clivus (30%) and the mobile spine (about 20%) [1]. 

Chordomas show a slow and locally infiltrative growth pattern. Immunohistochemically, the tumour cells express brachyury, pan-cytokeratin, EMA, S100-protein, vimentin, and INI-1 [4,5,6]. Histomorphologically, chordomas are classified by the WHO into three different types [7]: (i) conventional and chondroid chordomas demonstrate epithelioid, highly vacuolated cells amidst a myxoid matrix, also termed “physaliferous”; (ii) poorly differentiated chordomas are characterised by layers of epithelioid cells with non-physaliferous features and eosinophilic cytoplasm. Immunohistologically, these chordomas lose their INI-1 positivity and are more common in younger patients [8]; (iii) dedifferentiated chordomas appear partially conventional or chondroid with sarcomatously transformed components. The transformed components often lack the nuclear positivity for brachyury.

The first-line therapy of chordomas is radical en-bloc resection, which may be difficult due to the proximity of the chordoma to crucial structures, e.g., arteries and nerves. If tumour-free surgical margins are not achievable, careful resection followed by radiotherapy promises the best prospects for long-term disease control [1,9]. Unfortunately, chemotherapy treatments achieve only limited success rates [10,11]. Although clinical studies using receptor tyrosine kinase inhibitors such as EGFR inhibitors, VEGF inhibitors, and PDGFR inhibitors show some positive effects, there is no approved targeted therapy for general use in chordomas [10,12]. 

Chordomas are characterised by their overexpression of the *TBXT* gene and sequentially relevant nuclear quantities of the gene product brachyury [13]. Brachyury was shown to be essential in chordoma cell lines, as knockdown experiments induced changes in the cell morphology and reduced proliferation rates [14].

Cyclin-dependent kinases (CDKs) are a group of proteins that regulate the cell cycle and cell proliferation. The dysregulation of CDK activity is a common feature in many types of cancer, making CDK inhibitors suitable as therapeutic targets. CDK inhibitors (particularly inhibitors of CDK7 and CDK9) have been shown to disrupt the autoregulatory landscape of brachyury, downregulate cellular brachyury levels in a limited number of chordoma cell lines, and reduce tumour growth in vivo [14,15]. Nonetheless, the role of Cyclin-dependent kinases in chordomas is not yet fully understood.

In this study, we describe the establishment of the new chordoma cell line U-CH22. The cell line was derived from the pleural effusion of a high-grade disseminated chordoma of a 78-year-old Caucasian female. It was classified as a poorly differentiated chordoma, according to the WHO. U-CH22 is stable for over fifty passages and has a very high proliferation rate. We used the CDK7 inhibitor THZ1 and the CDK1,2,5 and 9 inhibitor dinaciclib to impede *TBXT* transcription. It has been shown that chordoma cell lines from different localisations and with different progression statuses show distinct gene expression patterns and vulnerabilities [16]. For this reason, we included cell lines of primary, recurrent and metastasised chordomas from sacral and clival locations in this study. With this broad spectrum of chordoma cell lines, we tested the effect of CDK inhibitors on the amount of brachyury mRNA and brachyury protein.

## 2. Materials and Methods

### 2.1. Establishment of the Chordoma Cell Line U-CH22

A total of 800 mL of pleural effusion was distributed to 50 mL Falcon tubes. These tubes were centrifuged, the pellets resuspended in 6 mL of erythrocyte lysis buffer, centrifuged again, and finally resuspended in fresh culture medium. The cell suspension was transferred to T25 culture flasks to allow for the adherence of the cells.

The cells were cultured in a chordoma cell-recommended Iscove’s Modified Dulbecco’s Medium mixed medium (4 parts IMDM + 1 part RPMI 1640; Gibco, Life Technologies Limited, Paisley, UK) supplemented with 10% foetal bovine serum (Sigma-Aldrich, St. Louis, MO, USA), 2 mM of glutamine (Lonza, Basel, Switzerland), and 1% penicillin/streptomycin (Gibco, Life Technologies Limited, Paisley, UK). The cells were incubated at 37 °C in an atmosphere with 95% relative humidity and 5% CO_2_. For passaging, the confluent cells were washed with 1× Dulbecco’s phosphate-buffered saline (PBS) without calcium or magnesium (Gibco, Life Technologies Limited, Paisley, UK) and trypsinisation was performed with Trypsin-EDTA (0.25%) (Gibco, Life Technologies Limited, Paisley, UK).

### 2.2. Screening for Mycoplasma Contamination

Mycoplasma contamination was assessed using a PCR approach (95 °C for 10 min followed by 55 cycles at 95 °C, 50 °C, and 68 °C, and 10 min at 72 °C). The mycoplasma primers used were forward: 5′ CRC CTG RGT AGT AHR HWC AG 3′ and reverse: 5′ GCG GTG TGT ACA ARM CCC GA 3′.

### 2.3. Proliferation Measurement

The growth kinetics of the cell line were assessed via MTS-based cell proliferation assay as suggested in the manufacturer’s handbook (MTS Assay Kit, Abcam, Cambridge, UK). The cells were seeded into two 96-well flat-bottom culture plates with 2000 cells per well and observed for 7 days (*n* = 6 per time point). Formazan absorption was measured using a spectrophotometer (Microplate Spectrophotometer, BioTek Instruments Inc., Winooski, VT, USA) at wavelengths of 490 nm and 650 nm. 

For *TBXT* rescue experiments, U-CH22 cells were seeded in a 6-well format at ~90% confluency in a growth medium without antibiotics. The following day, 2 µg of expression vectors (empty vector or TBXT-ORF pCMV6 expression vector (Origene, Rockville, MD, USA)) were transfected using Lipofectamine 2000 (Thermo Fisher Scientific, Rockford, IL, USA) according to the manufacturer’s recommendations. After overnight incubation, the transfected cells were seeded in 96-well plates (2500 cells/well) and incubated in culture media containing rising concentrations of dinaciclib. After 4 days of treatment, cell viability was determined as described above.

### 2.4. Library Preparation and Next-Generation Sequencing

For library preparation, 40 ng of isolated DNA was used in conjunction with the Tumor Mutational Burden panel (Qiagen, Hilden, Germany, # DHS-6600Z) using the QIASeq protocol in accordance with the manufacturer’s instructions. The resulting library was quantified via Qubit measurement using the Qubit dsDNA High-Sensitivity Assay Kit (Thermo Fisher Scientific, Waltham, MA, USA) and the quality was checked using tape station (HS D1000 ScreenTape assay, Agilent, Santa Clara, CA, USA). Subsequently, the library was sequenced on a NovaSeq device (Illumina, San Diego, CA, USA) and the resulting fastq files were processed using the CLC Genomics Workbench version 23.0.3 (Qiagen, Hilden, Germany). Variants were filtered using the following parameters: reference allele: no, EUR 1000GENOMES-phase_3_ensembl_v106_hg38_no_alt_analysis_set: ≤ 1%, variant allele frequency: ≥ 3%. All remaining variants were manually curated using the BAM files in conjunction with the integrative genomics viewer (version 2.8.13) and classified using the dbSNP, ClinVar, OnkoKB, CKB, and Civic databases.

### 2.5. Short Tandem Repeat (STR) Analysis

Short tandem repeat analysis was performed using the GenomeLab Human STR Primer Set (AB SCIEX, Framingham, MA, USA) and the AmpliTaq Gold DNA-Polymerase (Applied Biosystems by Thermo Fisher Scientific, Rockford, IL, USA) in accordance with the manufacturer’s instructions.

The PCR was performed in a Sensoquest Labcycler 48 (SensoQuest GmbH, Göttingen, Germany). Subsequently, the PCR products were separated on the CEQ GeXP capillary electrophoresis system (Beckman Coulter, Brea, CA, USA) and analysed using the Gene marker software (v.2.2.0, Softgenetics, State College, PA, USA). 

### 2.6. Cell Lines

The chordoma cell lines used in this study were U-CH1, U-CH2, U-CH17PII, U-CH17M, U-CH22, U-CHCF365, UM-Chor1, MUG-Chor1, and MUG-CC1. Table 1 indicates the variety of the panel with respect to the tumour localisation, the progression status of the disease, and the age of the patients. 

### 2.7. CDK Inhibition

Cells were seeded into 6-well plates with 500 000 cells per well. The CDK7 inhibitor THZ1 (THZ1 2HCl, Selleckchem, Houston, TX, USA) and the CDK1,2,5,9 inhibitor dinaciclib (dinaciclib (SCH727965), Selleckchem, Houston, TX, USA)) were dissolved in DMSO (Roth, Karlsruhe, Germany) to 5 mM as stock for further dilutions.

The chordoma cells were treated for 24 h with these two inhibitors in concentrations ranging from 1 nM to 10 µM. A culture medium containing 0.1% DMSO was used for negative controls. Following either, total RNA or total proteins were isolated using the RNeasy Mini Kit (Qiagen, Hilden, Germany) or RIPA buffer (Thermo Fisher Scientific, Rockford, IL, USA), containing protease/phosphatase inhibitors (Halt Protease & Phosphatase Inhibitor, Single-Use Cocktail (100×) (Thermo Fisher Scientific, Rockford, IL, USA), respectively.

### 2.8. Quantitative Polymerase Chain Reaction (qPCR)

RNA was isolated via an RNeasy Mini Kit (Qiagen, Hilden, Germany) and transcribed to cDNA via the SuperScript IV First-Strand Synthesis kit (Thermo Fisher Scientific, Rockford, IL, USA). Both kits were used in accordance with the manufacturer’s instructions. qPCRs were performed in quadruplicate using the QuantiTect SYBR Green PCR Kit (Qiagen, Hilden, Germany) on the Rotor-Gene Q 5plex platform (Qiagen, Hilden, Germany).

Due to their stable expression levels in a variety of tissues, the housekeepers GAPDH and β-actin were used as the internal control. 

The following primers were used in the experiments: β-actin forward: 5′ CCA CCA TGT ACC CTG GCA TT 3′ and reverse: 5′ TCA GGA GGA GCA ATG ATC TTG A 3′; GAPDH forward: 5′ CCT GCA CCA CCA ACT GCT TA 3′ and reverse: 5′ TGG CAT GGA CTG TGG TCA TG 3′; and brachyury forward: 5′ GTG ACC AAG AAC GGC AGG AG 3′ and reverse: 5′ TAC TTC CAG CGG TGG TTG TC 3′.

The expression levels were calculated based on the 2^−ΔΔCT^ method.

### 2.9. Western Blot Analysis

For the total protein analysis, adherent cells were lysed in 10 µL/cm² of RIPA buffer (Thermo Fisher Scientific, Rockford, IL, USA) supplemented with 1% Halt Protease and Phosphatase Inhibitor, Single-Use Cocktail (100×) (Thermo Fisher Scientific, Rockford, IL, USA). The lysate was shock-frozen in liquid nitrogen twice and finally centrifuged for 10 min at 4 °C with 20,817× *g*.

After measuring the protein concentrations via the Pierce BCA protein Assay Kit (Thermo Fisher Scientific, Rockford, IL, USA), the protein concentrations were balanced with RIPA buffer. Western blot gels (NuPage 4–12% Bis-Tris Gel, Thermo Fisher Scientific, Rockford, IL, USA) were loaded with the samples for SDS-Page and then electro-blotted onto nitrocellulose membranes (Amersham Protram 0.2 µm NC Nitrocellulose Blotting Membrane, Cytiva, Marlborough, MA, USA).

The primary antibodies used were brachyury (D2Z3J, Cell Signaling, Danvers, MA, USA) as well as two housekeepers: GAPDH (14C10, Cell Signaling, Danvers, MA, USA) and beta-actin (Clone AC-74, Sigma Aldrich, St. Louis, MO, USA). As secondary antibodies anti-mouse (Goat IgG (H+L), HRP conjugate, Invitrogen, Carlsbad, CA, USA) and anti-rabbit (A0545, whole molecule peroxidase antibody produced in goat, Sigma Aldrich, St. Louis, MO, USA) were used. The membranes were developed in a C-DiGit^®^ Blot Scanner (Li-Cor, Lincoln, NE, USA) after a 5 min incubation in SuperSignal West Pico PLUS Chemiluminescence-Substrate (Thermo Fisher Scientific, Rockford, IL, USA). All Western blots were performed in triplicate.

### 2.10. Immunocytochemistry and Immunohistochemistry

For immunocytochemical analyses of cultured cells, paraffin cell blocks were produced using standard protocols. In short, chordoma cells were trypsinised, pre-fixed in 100% ethanol-containing haematoxylin (Dako, Glostrup, Denmark), and embedded in paraffin. For immunohistochemistry, tissue samples were fixed in formaldehyde and embedded in paraffin.

Sections were cut with a thickness of 2 µm. For immunochemistry, the sections were dewaxed and antibody-specific antigen retrieval methods (see Table 2) were applied. The used antibodies were previously validated using several positive and negative controls to ensure the specific staining of the antigen. 

The pretreated slides were incubated for 30 min with the diluted antibodies and treated with the avidin–biotin-complex method using the Dako REAL Detection System, Alkaline Phosphatase/RED (Dako, Glostrup, Denmark). 

### 2.11. Fluorescence In Situ Hybridisation (FISH)

Fluorescence in situ hybridisation (FISH) was performed on formalin-fixed, paraffin-embedded (FFPE) sections using a Zyto Light SPEC SMARCB1/22q12 Dual Color Probe (ZytoVision GmbH, Bremerhaven, Germany) and standard procedures. Sections were deparaffinised via descending ethanol series. For the pretreatment, the slides were placed in a steamer at 98 °C; citrate buffer (pH 6)/1 mM EDTA. The slides were digested with a pepsin solution (Zytomed Systems, Berlin, Germany) at 37 °C, pretreated with a saline–sodium–citrate solution at 37 °C and dehydrated in an ascending ethanol series. Dried, dehydrated slides were treated with the probe, denatured at 75 °C, and hybridised overnight at 37 °C. Slides were washed and counterstained with DAPI, then analysed with an Axio Imager.A2m microscope (Zeiss, Oberkochen, Germany), appropriate fluorescent filters, and imaging software (Ikaros Neon v.6.3.5, MetaSystems, Altlussheim, Germany). A total of 100 interphase nuclei were analysed for each specimen. A total of 15% of the nuclei demonstrating signal loss were defined as a cut-off for the deletion of the *SMARCB1* gene locus. 

## 3. Results

### 3.1. Features of U-CH22

#### 3.1.1. Case Report

A 78-year-old female presented with a large tumour in the left thigh (Figure S1A–G). Histologically, the tumour exhibited epithelioid cytology with few physaliferous cells, but a chordoma-typical immunohistochemical profile (positive for brachyury, pan-cytokeratin, EMA, and focal positivity for S100-protein; Figure S2), leading to the diagnosis high-grade chordoma (FNCLCC Score: 3 + 2 + 0 = 5 = G2, high-grade). 

In addition to the tumour in the thigh, smaller lesions were found in the patient’s ilium and sacrum. The patient was treated with imatinib, radiotherapy, and ultimately olaparib. The chordoma, however, progressed and pan-metastasised, including a malignant pleural effusion. Metastases of the chordoma were found in the sacrum, ilium, clavicle, lungs, peritoneum, lymph nodes, adrenal gland, and thyroid. All these lesions progressed in growth over a period of 2 months (Figure S3A–J). 

The chordoma showed aspects of a poorly differentiated chordoma (nests of epithelioid cells and scattered cytoplasmatic vacuoles reminiscent of signet-ring cells [17]) and destructive infiltrations of the bone and adjacent soft tissue (Figure S1B–G) as well as rapid tumour growth of all lesions (Figure S3A–J). Thus, comparative INI-1 stainings and *SMARCB1* fluorescence in situ hybridisations (FISHs) of the primary tumour, the lymph node metastasis, and the pleural effusion were performed. Although the FISH analysis excluded a *SMARCB1* gene deletion, the lack of INI-1 nuclear immunocytochemical staining favoured a loss of *SMARCB1* expression (Figure S7(A3–C3)). This classified the chordoma as a rare subtype of a poorly differentiated chordoma, exhibiting a deficiency in *SMARCB1* expression [18]. 

The largest tumour mass was found in the left thigh. Comparing the proliferation rates (determined with KI-67 indices) of this lesion, a metastasis of an inguinal lymph node, the tumour cells found in the pleural effusion and the consequential established cell line U-CH22 indicate an increase in proliferation rates during tumour progression (Figure S4).

#### 3.1.2. Cell Culture

**Figure 1:**
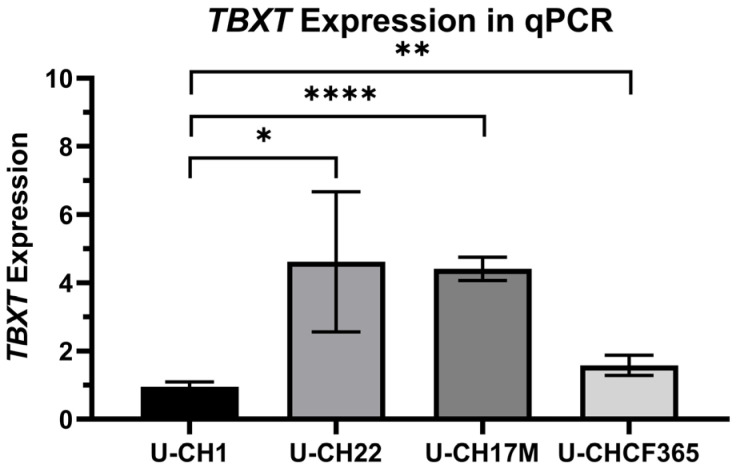
*TBXT* expression in the 4 different chordoma cell lines U-CH1, U-CH17M, U-CH22, and U-CHCF365. Expression levels were normalised to the expression level of the U-CH1 cell line (*n* = 3); * indicates significance of the differences (T-tests; * = *p* < 0.0.5; ** = *p* < 0.01; **** = *p* < 0.0001).

U-CH22 cells were passaged for >50 population doublings. No signs of senescence or growth speed reduction were seen, implying the immortality of the tumour cells in vitro. The cells showed a moderately heterogenous morphology, mostly with an epithelial appearance when reaching 100% confluency (Figure S5A,B). The chordoma-typical physaliferous appearance was only present in a minority of the chordoma cells. Furthermore, the proliferation speed of the cells was atypically high. MTS assays revealed a population doubling time of about two days (Figure S5C), making U-CH22 one of the fastest-growing chordoma cell lines compared to other chordoma cell lines listed by the Chordoma Foundation [19]. To verify U-CH22 as a true chordoma cell line, immunocytochemistry was used to confirm the positivity of the cells for brachyury, vimentin, EMA, S100 protein, and pan-cytokeratin (Figure S6). The cells clearly expressed these markers except for the S100 protein. Here, only a subpopulation of the cells was positive and in line with other chordoma cell lines. The KI-67 index was >80%, confirming the short population doubling time of U-CH22. An expression analysis via qPCR revealed a *TBXT* expression ~4.6-fold compared to the common reference U-CH1 (see Figure 1). 

To further characterise the cell line, immunocytochemical staining for INI-1 and p53 was performed. INI-1 was negative (nuclear), implying a common loss of *SMARCB1* (encoding for INI-1) expression (Figures S6G and S7). The p53 staining was negative. Deep sequencing revealed that U-CH22 harbours critical mutations in the *TP53* gene, a possible splice site disruptive mutation (c.69+1G>C) and a mutation causing a premature stop codon (c.538G>T), potentially fostering the tumour’s aggressiveness and proliferation speed. These mutations most probably explain the p53 negativity of U-CH22 cells.

To confirm the origin of the cell line, short tandem repeat (STR) analyses were performed using DNA isolated from the archived tumour material of the patient and from cultured U-CH22 cells. The resulting profiles matched perfectly in all tested markers (Table S1).

### 3.2. THZ1 and Dinaciclib Reduce TBXT Expression on mRNA Levels in U-CH22

CDK inhibitors have been shown to downregulate cellular brachyury levels in a limited number of chordoma cell lines [14]. We wanted to test how CDK inhibition influences the *TBXT* expression in the newly established cell line U-CH22. Since U-CH22 was derived from an extremely aggressive chordoma resistant to imatinib and olaparib, we tested whether this recent approach might also be able to influence the *TBXT* expression in highly progressed late-stage chordoma.

**Figure 2:**
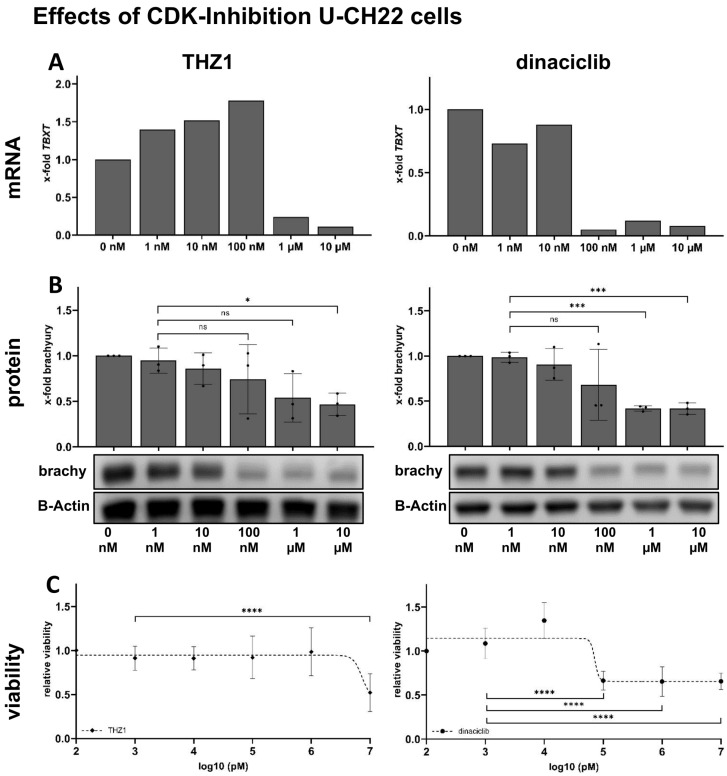
Effects of CDK7/CDK9 inhibition on the cell line U-CH22. (**A**) *TBXT* mRNA levels determined via qPCR are drastically reduced in U-CH22 treated with concentrations ≥1 µM of THZ1 and ≥100 nM of dinaciclib (*n* = 1 biological replicate, *n* = 4 technical replicates). (**B**) Dose-dependent reduction in brachyury levels in THZ1/dinaciclib-treated U-CH22 cells (*n* = 3 biological replicates, 1 Western blot result is shown exemplarily). (**C**) Viability measurements of U-CH22 after treatment with increasing concentrations of THZ1 and dinaciclib (*n* = 6). * Indicates significance of the differences (T-tests; ns = *p* > 0.05; * = *p* < 0.05; *** = *p* < 0.001; **** = *p* < 0.0001).

To test the effects of CDK inhibitors on the *TBXT* expression on mRNA levels, U-CH22 cells were incubated with rising concentrations of THZ1 and dinaciclib. mRNA levels of *TBXT* were drastically reduced, with IC50 values being 878.4 nM for THZ1 and 32.0 nM for dinaciclib. The maximal reduction in *TBXT* mRNA levels by about -90% was reached using 1 µM of THZ1 or 100 mM of dinaciclib, respectively (Figure 2A).

### 3.3. THZ1 and Dinaciclib Reduce TBXT Expression on Protein Levels in U-CH22

Next, we assessed whether the reduced *TBXT* message is comparably reflected in the brachyury protein levels. The Western blot detection of brachyury, after treating U-CH22 cells with rising concentrations of THZ1 or dinaciclib for 24 h, showed a clear-cut reduction in brachyury protein.

The effective dynamic of the inhibitors was similar regarding the brachyury protein levels. Trends of reduced brachyury levels were seen using 1 µM of THZ1 and 100 nM of dinaciclib. Significantly reduced protein levels to a maximum of 40–50% remaining brachyury were reached at 10 µM of THZ1 and 1 µM of dinaciclib, respectively. The maximum concentration of 10 µM of THZ1 reduced brachyury levels in U-CH22 cells to 47% (SD +/− 12.2%) of the control value. A total of 10 µM of dinaciclib reduced brachyury in U-CH22 to 42% (SD +/− 6.3%) of the corresponding control (Figure 2B). 

By measuring brachyury levels, the IC50 value for THZ1 was 85.3 nM and the IC50 for dinaciclib was 76.6 nM in U-CH22 cells.

### 3.4. THZ1 and Dinaciclib Reduce the Viability of U-CH22

Knowing that THZ1 and dinaciclib reliably reduce the *TBXT* expression in U-CH22, we tested whether the reduced levels of the chordoma key player brachyury have an impact on the cells’ viability. THZ1 treatment did not affect the viability of U-CH22 cells until the highest concentration (10 µM) was reached. At this maximal concentration, the viability was reduced to about 50% compared to cells treated with the vehicle control (0.1% DMSO). Dinaciclib decreased the number of viable cells to about 60%, starting with concentrations of 100 nM. The amounts of measured brachyury did not decrease further at higher dinaciclib concentrations (Figure 2C). The dinaciclib treatment of U-CH22 cells, previously transfected with a *TBXT*-ORF-myc-expressing vector, did not rescue the cells’ viability (Figure S9), implicating that the viability reduction after CDK7/9 inhibition may not solely result from the reduction in brachyury levels but also from the impact on other essential super-enhancer-regulated genes. 

### 3.5. THZ1 and Dinaciclib Reduce the TBXT Message in Four Cell Culture Models of Progressed Chordoma

Since the CDK inhibitors THZ1 and dinaciclib were able to reduce *TBXT* expression in the model of an ultimately progressed chordoma (U-CH22), we next tested whether this might be an individual effect or whether additional models of progressed chordoma respond to these inhibitors as well. To address this question, we chose U-CH1 as the best-studied model of a recurrent sacral chordoma, U-CH17M, a model derived from the metastasis of a sacral chordoma and U-CHCF365 to include a model of a recurrent and metastasised paediatric clival chordoma.

To determine the baseline *TBXT* expression, mRNA levels were measured using RT-qPCR and normalised to the expression levels of U-CH1 (Figure 1). The three chordoma cell lines U-CH22, U-CH17M, and U-CHCF365 were shown to have a higher baseline *TBXT* expression than U-CH1 (2–4.5-fold). 

**Figure 3:**
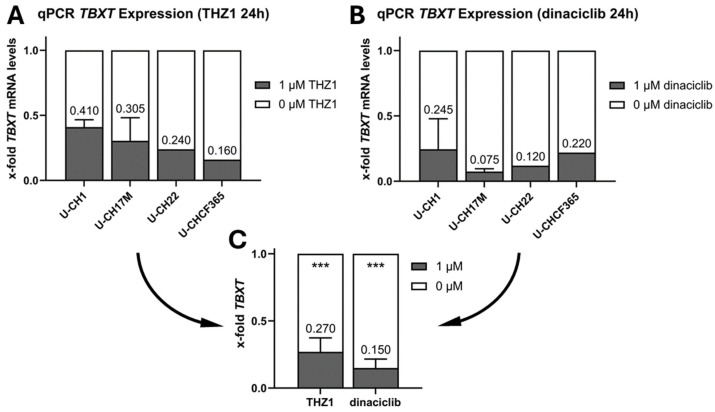
*TBXT* mRNA levels of U-CHCF365, U-CH22, U-CH1, and U-CH17M after inhibition with 1 µM of THZ1 (**A**) or dinaciclib (**B**) for 24 h (U-CH22+U-CHCF365: *n* = 1; U-CH1+U-CH17M: *n* = 2); (**C**) comparison of *TBXT* mRNA reduction with THZ1 and dinaciclib across chordoma cell lines; * indicates significance of the differences (T-tests; *** = *p* < 0.001).

To assess whether CDK inhibition influences the *TBXT* expression on mRNA levels, the cell lines U-CH1, U-CH17M, U-CH22, and U-CHCF365 were incubated with rising concentrations of THZ1 and dinaciclib. mRNA levels were drastically reduced in all cell lines following the same effect dynamics as were initially seen for U-CH22. At a concentration of 1 µM, THZ1 reduced the *TBXT* mRNA levels of the 4 tested cell lines to levels between 16% (U-CHCF365) and 41% (U-CH1) of the corresponding vehicle control-treated cells (Figure 3A). Dinaciclib showed a stronger effect with a concentration of 1 µM. We detected reductions in *TBXT* mRNA to levels between 7.5% and 25% of the control (Figure 3B). When treated with even higher concentrations of 10 µM of THZ1, the *TBXT* mRNA levels dropped to around 11% across the cell lines. However, with these high inhibitor concentrations, the viability of the cells was significantly reduced, and adherent cells detached from the surface.

The calculated IC50 values for THZ1 were 842.9 nM in U-CH1, 886.7 nM in U-CH17M, 878.4 nM in U-CH22, and 51.8 nM in U-CHCF365. For dinaciclib, the IC50 values were 6.1 nM in U-CH1, 10.4 nM in U-CH17M, 32.0 nM in U-CH22, and 2.4 nM in U-CHCF365. 

### 3.6. THZ1 and Dinaciclib Reduce Brachyury Levels in Nine Chordoma Cell Lines

To further substantiate our findings, we increased the panel of investigated chordoma cell lines to a total of nine cell lines covering the main locations and all stages of tumour progression. U-CH1, U-CH2, and MUG-Chor1 are cell lines that originate from middle-aged patients with a recurrent chordoma tumour that manifested at the patients’ sacrum. U-CH17PII and U-CH17M are cell lines that originate from a primary chordoma at the sacrum and a corresponding metastasis of the same patient. UM-Chor1 and MUG-CC1 were derived from primary clival chordomas and U-CHCF365 was established from the tumour tissue of a patient harbouring a recurrent and metastatic clival chordoma. The newly established cell line U-CH22 is the model of a high-grade pan-metastasised chordoma derived from pleural effusion cells. 

Independent of the localisation or progression status of the originating tumour, a significant reduction in brachyury levels was detected in all chordoma cell lines after 24 h of treatment with the CDK inhibitors THZ1 and dinaciclib. However, the level of brachyury reduction as well as the IC50 for THZ1 and dinaciclib varied between the cell lines. 

**Figure 4:**
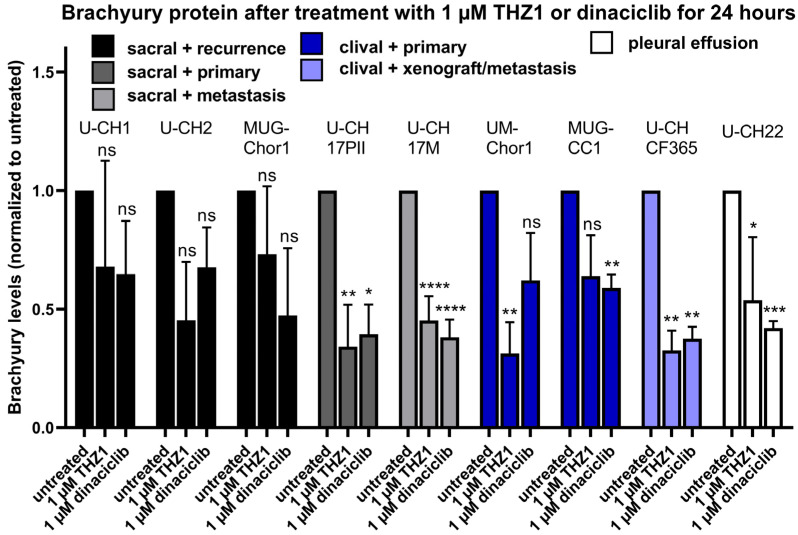
Relative amounts of brachyury protein in nine chordoma cell lines after treatment with 1 µM of THZ1 or 1 µM of dinaciclib for 24 h. The colour indicates the tumour location and the progression status of the disease (*n* = 3–6), error bars indicate mean + SD, and * indicates the significance of the differences to the untreated group (one sample t-test; ns = *p* > 0.05; * = *p* < 0.05; ** = *p* < 0.01; *** = *p* < 0.001; **** = *p* < 0.0001).

The nine cell lines were treated with THZ1 and dinaciclib for 24 h with concentrations ranging from 1 nM to 10 µM. The most prominent brachyury protein reduction (a downregulation to 31% protein compared to the vehicle control) was achieved with 1 µM THZ1 in the cell line UM-Chor1. In general, both CDK inhibitors were effective in all tested chordoma cell lines and reduced the amount of brachyury protein to a mean of about 50% (Figure 4, for exemplary Western Blots, see Figure S8). 

The molarity of CDK inhibitors necessary to reach the half-maximal reduction in brachyury levels varied between the cell lines. For THZ1, the IC50 values predominantly ranged between 100 nM and 1 µM, roughly matching the concentration found to be half-maximal effective for *TBXT* message reduction (Table 3). Only U-CHCF365 and MUG-CC1 tended to be more susceptible to THZ1-induced brachyury reduction, showing IC50 values of 11 nM and 22 nM, respectively. The calculated IC50 concentrations for dinaciclib were found to be between 8 nM and 87 nM, resembling a range comparable to that previously determined for *TBXT* mRNA reduction. 

Taken together, CDK inhibition (predominantly CDK7 and CDK9 inhibition) is a suitable option for reducing *TBXT* expression in chordoma cell lines.

## 4. Discussion

We describe the establishment of a novel chordoma cell line from the pleural effusion of a patient suffering from a poorly differentiated, pan-metastasised, high-grade chordoma. In our experiments, the cell culture parameters of U-CH22 were a good match to the other chordoma cell lines, rendering U-CH22 a proper model for chordoma. Since multiple metastases throughout many different patient tissues and locations are rare in this per se very rare disease, U-CH22 is an interesting and remarkable extension to the panel of chordoma in vitro models available. The most striking feature of U-CH22 is its high proliferation index and its insensitivity to lower cell densities. Chordoma cell lines mostly have low proliferation rates with population doubling times of up to 2 weeks. Furthermore, the in vitro growth of many chordoma cell lines strongly depends on cell densities of >50% confluency to ensure consistent proliferation. This hampers the cultivation and expansion of the cells and impedes in vitro assays based on proper and consistent cell growth, e.g., wound-healing assays or growth inhibitor testing. 

The morphology of the primary tumour of U-CH22 is not completely conventional and the immunohistochemical profile of this tumour was that of a poorly differentiated chordoma as seen in the negative INI-1 stainings; however, fluorescence in situ hybridisation revealed no loss of the *SMARCB1* gene locus. Therefore, in this case of a poorly differentiated chordoma, the loss of *SMARCB1* expression was due to gene silencing rather than genomic loss of the gene locus, a mechanism already described for other poorly differentiated chordomas [18]. The aggressive and infiltrative growth as well as the wide metastatic spread further matched the diagnosis of poorly differentiated chordoma. Preserved *SMARCB1* status in poorly differentiated chordoma is very rare but not unprecedented [18]. Nevertheless, it must be noted that upon diagnosis, the patient was 78 years old and not under 30, as is more often the case for poorly differentiated chordomas [7].

RT-qPCR-based *TBXT* expression analyses showed that CDK inhibitors THZ1 and dinaciclib decreased *TBXT* mRNA levels in four chordoma cell lines in a dosage-dependent manner. A total of 1 µM of THZ1 decreased the *TBXT* mRNA levels to about 30% overall in the four cell lines, and 1 µM of dinaciclib decreased the overall mRNA levels to about 20%. This outcome might be explained by the fact that, in contrast to dinaciclib, which inhibits several Cyclin-dependent kinases (specifically CDK1, CDK2, CDK5, and CDK9), THZ1 predominantly inhibits CDK7. The exact mechanism of how CDK inhibitors are able to decrease *TBXT* expression in chordoma cells is not entirely understood. Some studies suggest that the CDK7 might be part of a transcriptional condensate that plays a crucial role in the autoregulation of brachyury [15]. Transcriptional CDKs like CDK9 and CDK7 are also often located near enhancers of genes [20,21]. This was tested by Sharifnia et al. in UM-Chor1 cells, where *TBXT* expression was separated from the regulatory super-enhancers via transduction with an expression vector encoding *TBXT* under the control of an exogenous promoter. In these cells, the ectopic expression levels of brachyury protein were not significantly reduced via inhibition with THZ1 compared to endogenous brachyury levels, which were clearly reduced by the treatment [14]. Sharifnia et al. were also able to show that the CDK inhibitor THZ1 is able to inhibit the RNA polymerase II (POLR2A), potentially by reducing the phosphorylation of the C-terminal domain of POLR2A (specifically Serin 2, 5, and 7) and the total amount of POLR2A. This effect starts for THZ1 between 50 and 500 nM, fitting quite well to the effective concentrations described in our manuscript. Furthermore, the authors were able to show that the global impact on transcription is stronger for genes under the control of super-enhancers (such as *TBXT*). In addition to the antiproliferative effect on chordoma cells, increased caspase-3/7 activity was measured after THZ1 treatment, implicating the induction of apoptosis [14]. To date, it has not yet been convincingly shown whether this was induced by brachyury knockdown or *TBXT*-independent pathways. Thus, more research is needed to fully understand the function of CDKs in chordomas.

Using Western blot analyses, we confirmed that the CDK inhibitors THZ1 and dinaciclib reduced the amount of brachyury protein in a total of nine chordoma cell lines, including U-CH22. These findings match recent studies conducted on the chordoma cell lines MUG-Chor1, UM-Chor1, JHC7, and U-CH1, showing a distinct reduction in brachyury levels after treatment with high concentrations of these inhibitors [14]. Interestingly, brachyury levels could not be reduced below 30–60% in this study (depending on the individual cell line), although mRNA levels were altered to about 10% using higher concentrations of THZ1 or dinaciclib. This implicates intracellular rescue mechanisms expanding the protein half-life time or enhanced *TBXT* mRNA translation connected to residual brachyury amounts. The underlying mechanism of this minimum brachyury level preservation in chordoma urgently needs to be further explored in order to find options for the inhibitor-guided complete obliteration of brachyury within chordoma cells. 

U-CHCF365 and MUG-CC1 cells (representing two-thirds of cell lines of clival origin within the panel) showed an approximately 15- to 30-fold higher susceptibility to THZ1 than the other cell lines (median IC50 308 nM). Although the low number of clival chordoma cell lines narrows the statistical validity of the assumption that clival chordomas might be particularly prone to CDK7 inhibition, at least no specific resistance linked to the location of the tumour can be postulated.

In MTS-based viability assays with THZ1 and dinaciclib, we demonstrated a decrease in the viability of U-CH22 chordoma cells to a maximum of about 50% and 60%, respectively, using the highest concentrations of 10 µM of the inhibitors. This matches the findings of Sharifnia et al. [14] for the chordoma cell lines UM-Chor1, JHC7, U-CH1, and MUG-Chor1. Here, using even higher inhibitor concentrations of up to 32 µM, the viability rates dropped to about 10–30%. To figure out if the viability reduction after CDK7/9 inhibition is primarily due to the reduction of brachyury levels, we performed viability measurements using dinaciclib-treated cells, previously transfected with a *TBXT* expression vector, to test if the viability can be rescued at least in parts via the overexpression of *TBXT*. Overexpressing *TBXT* did not lead to an increase in cell viability. However, we were able to show that the levels of ectopic brachyury were not affected by the inhibitor treatment, consistent with previous results showing that THZ1 treatment in chordoma cells has no effect on the expression of transfected *TBXT*-ORF vectors [14]. Although the validity of the experiments has limitations due to treatment-related adversities (e.g., high baseline toxicity in the very sensitive chordoma cells or time-dependent reduction in the expression of the transfected *TBXT*-ORF vector), it can be assumed that brachyury level reduction after dinaciclib treatment may not be the sole factor for the detected viability reduction. Sheppard et al. [15] showed that the further degradation of remaining brachyury using specific degrons did not additionally reduce cell viability in chordoma cells after THZ1 treatment, substantiating this idea as well. Although additional studies have to be performed, there is growing evidence suggesting that the decrease in viability observed in chordoma cells after CDK7/9 inhibition may be attributed not solely to the reduction in brachyury but also to the global expression reduction, especially in super-enhancer-regulated genes. Nonetheless, CDK7/9 inhibition seems to provide an opportunity to target the cell viability of chordoma cells. 

Several CDK inhibitors have been approved for the treatment of cancer, such as palbociclib, ribociclib, and abemaciclib. These inhibitors have shown a clinical benefit in combination with other cancer therapies, particularly in breast carcinoma [22]. CDK7 and CDK9 were shown to reduce the viability of chordoma cells through a genome-scale CRISPR/Cas9 approach as well as the large-scale testing of >450 small molecules [14]. Because of these findings, we chose the CDK inhibitors dinaciclib (CDK1, CDK2, CDK5, and CDK9) and THZ1 (CDK7) for our experiments. Other CDK inhibitors like palbociclib (CDK4 and CDK6) were also able to reduce chordoma cell line proliferation but did not result in any significant reduction in brachyury. Inhibition experiments using CDK8 and CDK19 inhibitor BRD6989 did not alter cellular viability [14,23]. Further investigation is required to bridge the significant knowledge gap regarding how CDK inhibitors impact cellular mechanisms in chordomas, as well as to identify which CDK inhibitors exhibit efficacy against this disease. 

THZ1 stands out as one of the most extensively examined covalent inhibitors of CDK7. Preclinical investigations have consistently revealed robust anti-tumour effects across a range of cancer types, for example osteosarcoma [24], multiple myeloma [25], and breast carcinoma [26]. SY-1365, a modified variant of THZ1 and a CDK7 inhibitor, started its phase I clinical trial for the treatment of ovarian and breast carcinomas in 2017 [27,28]. 

Dinaciclib has been studied in combination with other therapeutics such as heat-shock protein inhibitors [24] and TNF-related apoptosis-inducing ligand (TRAIL) [29] to enhance its anticancer effects. Its broader target profile with four different inhibited CDKs makes it more amenable to combination approaches, allowing synergistic effects in cancer treatment. In osteosarcomas, the combination of dinaciclib and heat-shock protein 90 inhibitors reliably induced apoptosis in vitro [24]. Dinaciclib has advanced further in clinical development compared to THZ1. In September 2023, dinaciclib progressed to a phase III clinical trial, demonstrating significant efficacy in lung carcinoma, breast carcinoma, multiple myeloma, and chronic lymphocytic leukaemia [25,26,30]. The advanced stage of clinical development suggests that it shows promise in terms of safety and efficacy in humans. 

One potential approach to enhancing the anti-tumour effects of CDK inhibitors, particularly by keeping toxicity to a minimum, might be the combination with other target classes. As mentioned above, dinaciclib has successfully been combined with a number of other therapeutics in a variety of cancers, but not yet in chordomas. Other potential target classes for the combination with CDK inhibitors could be EGFR/ERBB2 inhibitors or p300 inhibitors, which have previously been reported as having antiproliferative effects on chordomas [31,32]. Nonetheless, further research is needed to improve the efficacy and tolerability of CDK inhibitors and identify novel therapeutic targets.

## 5. Conclusions

We describe the testing of two different CDK inhibitors on a large cohort of primary, recurrent, metastasis-derived and paediatric chordoma cell lines as well as the newly established cell line U-CH22 derived from a high-grade disseminated chordoma. The CDK inhibitors THZ1 and dinaciclib significantly reduced the amount of the essential protein brachyury in all chordoma cell lines, ultimately reducing cell viability in the new cell line U-CH22.

## Figures and Tables

**Table 1:** List of cell lines used in our experiments.

Name	Species	Tumour	Localisation	Status	Patient Age	Origin
U-CH1	Human	Chordoma	sacral	recurrence	56	University Hospital Ulm (Germany)
U-CH2	Human	Chordoma	sacral	recurrence	72	University Hospital Ulm (Germany)
U-CH17PII	Human	Chordoma	sacral	primary	38	University Hospital Ulm (Germany)
U-CH17M	Human	Chordoma	sacral	metastasis	38	University Hospital Ulm (Germany)
U-CH22	Human	Chordoma	pleural effusion	recurrence + pan-metastasised	78	University Hospital Ulm (Germany)
U-CHCF365	Human	Chordoma	clival	Xenograft model of metastasis	<20	University Hospital Ulm (Germany)
UM-Chor1	Human	Chordoma	clival	primary	66	Michigan University (USA)
MUG-Chor1	Human	Chordoma	sacral	recurrence	57	Graz University (Austria)
MUG-CC1	Human	Chordoma	clival	primary	72	Graz University (Austria)

**Table 2:** Antibodies used for immunohistochemical analyses of the new chordoma cell line.

Antibody Name	Manufacturer (Reference No.:)	Species	Dilution	Pretreatment
brachyury	Abcam (ab209665)	rabbit	1:4000	Steamer; in EDTA pH 8
CDK4	Zytomed (A03-61096)	mouse	1:50	Steamer; in EDTA pH 8
CDK6	LS Bio (LS-B5388)	mouse	1:100	Pressure cooker
EMA (Clone E29)	Dako (M0613)	mouse	1:500	Pronase
INI-1 (Clone 25/BAF47)	BD Bioscience (612111)	mouse	1:50	Pressure cooker
KI-67 (Clone MIB-1)	Dako (M7240)	mouse	1:200	Pressure cooker
p53 (Clone DO-7)	Dako (M7001)	mouse	1:400	Microwave; in citric acid pH 6.1
pan-cytokeratin (Clone CK AE1/AE3)	Dako (M3515)	mouse	1:100	Pronase
S100 (Clone Polyclonal)	Dako (GA504)	rabbit	1:1000	Pronase
vimentin (Clone Vim 3B4)	Dako (M7020)	mouse	1:300	Microwave; in citric acid pH 6.1

**Table 3:** Molarity of CDK inhibitors THZ1 and dinaciclib necessary to reach the half-maximal amount of brachyury (IC50) in 9 chordoma cell lines.

Molarity of CDK Inhibitors to Reach the Half-Maximal Amount of Brachyury Protein		
	THZ1 [nM]	Dinaciclib [nM]
U-CH1	979	10
U-CH2	272	8
U-CH17M	333	11
U-CH17PII	918	10
U-CH22	132	83
U-CHCF365	11	11
UM-Chor1	308	11
MUG-Chor1	326	10
MUG-CC1	22	87

## Data Availability

All data are available upon request.

## Supplementary Materials

The following supporting information can be downloaded at: https://www.mdpi.com/article/10.3390/diagnostics14101028/s1, Table S1: Short tandem repeat analysis of U-CH22 and the corresponding tumour tissue. Figure S1: Symptomatic major lesion, contrast-enhanced CT, bone windowing. Figure S2: Morphology and immunohistochemistry of the primary tumour. Figure S3: The progression of the lesions over time, contrast-enhanced CT axial sections. Figure S4: Comparison of proliferation rates of the primary chordoma and the ultimate metastases. Figure S5: Phase contrast picture of cultured U-CH22 cells at about 50% confluence (A) and 100% confluence (B); growth curve of U-CH22 (C). Figure S6: Immunocytochemistry of U-CH22 at 40× magnification. Figure S7: Morphology and immunohistochemistry: primary chordoma (A), pleural effusion, (B) and U-CH22 cell line (C). Figure S8: Exemplary Western Blots used to generate Figure 4. Figure S9: Effects of *TBXT* overexpression in dinaciclib-treated U-CH22 cells.
