# Supplementary material for: Impact of CDK Inhibitors on TBXT Expression in Chordoma Cell Lines Including the First Stable Cell Line of a High-Grade Chordoma
Source: Diagnostics (Basel). 2024 May 16;14(10):1028. doi: 10.3390/diagnostics14101028 (PMC11120607; doi:10.3390/diagnostics14101028)
Supplement: Supplementary file 1 [file diagnostics-14-01028-s001.zip › diagnostics-2940572-supplementary.pdf]

Supplementary Figures and Tables

Table S1. Short tandem repeat analysis of U-CH22 and the corresponding tumour tissue.

Markers	U-CH22 cell line	U-CH22 tumour tissue
Amelogenin	X	X
D13S317	8; 10	8; 10
D7S820	8	8
D16S539	9; 12	9; 12
Penta E	10; 20	10; 20
TH01	9; 9,3	9; 9,3
D18S51	15; 16	15; 16
D3S1358	(15) 16	15; 16
D8S1179	9; 13	9; 13
TPOX	9; 11	9; 11
CSF1PO	11; 12	11; 12
Penta D	9	9

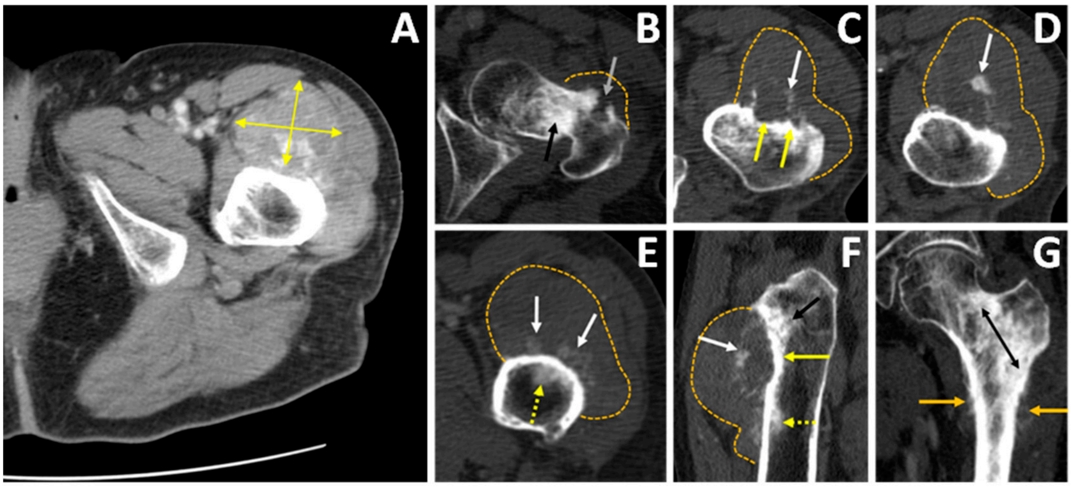

Figure S1. Symptomatic major lesion: a large, aggressive tumour mass, residing on the superficial (periosteal) aspect of the anterior proximal left femur, spreading superficially alongside the anterior periosteal surface of the proximal femur, transaxial section, contrast-enhanced CT, soft-tissue windowing (A); contrast-enhanced CT, bone windowing, transaxial sections at different levels of the proximal femur (B-E); contrast-enhanced CT, bone windowing, sagittally and coronally reformatted sections (F-G). Markings: tumour mass (orange dashed line); intramedullary reactive sclerosis or osteoblastic tumour proliferation (black arrows); cortical destruction (grey arrow); superficial cortical erosions (yellow arrows) with nearby and distant tumoural calcifications (white arrows); intramedullary tumour invasion (dashed yellow arrows); spiculated periosteal reactions (orange arrows).

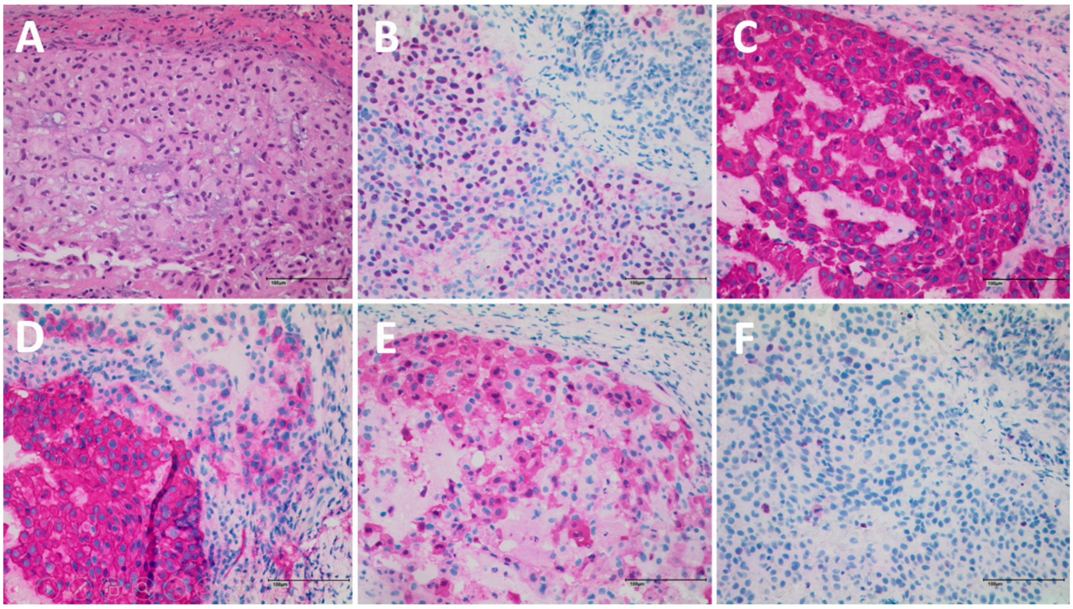

Figure S2. Morphology and immunohistochemistry of the primary tumour. A) H/E staining, (B) brachyury, (C) pan-cytokeratin, (D) EMA, (E)S100, and (F) KI-67; bar represents 100 µm.

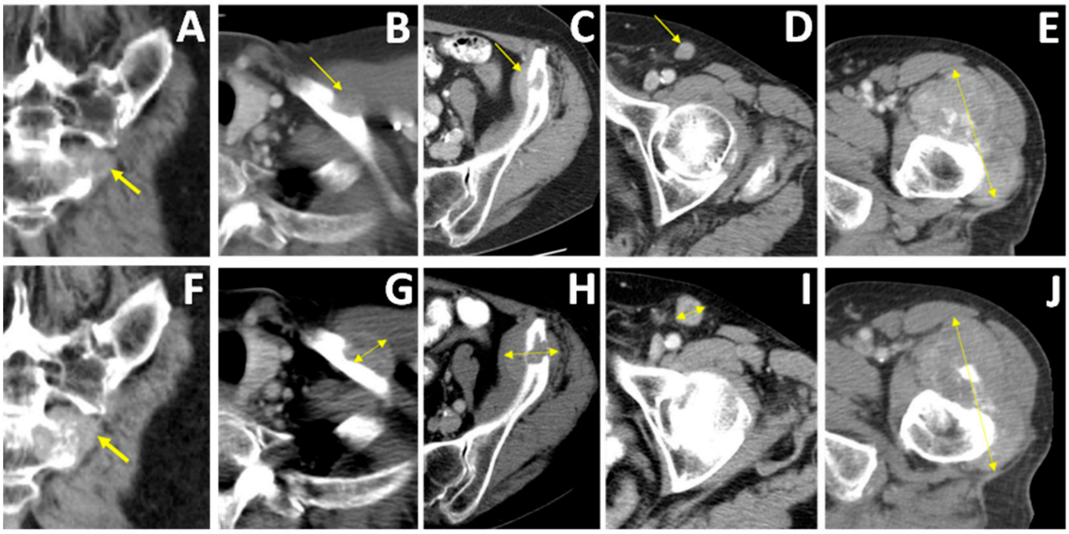

Figure S3. The progression of the lesions (yellow arrows) over time: contrast-enhanced CT axial sections, obtained at two consecutive time points (lower-row CT conducted 2 months after upper-row CT); left paramedian lower sacral mass at the level S4-S5, slight tumour growth (A+F); anterior shaft left clavicle (B+G); anterior part left iliac wing (C+H); left inguinal metastatic lymphadenopathy (D+I); index lesion of the proximal left femur (E+J); there is another focal left-sided iliac lymphadenopathy (not shown).

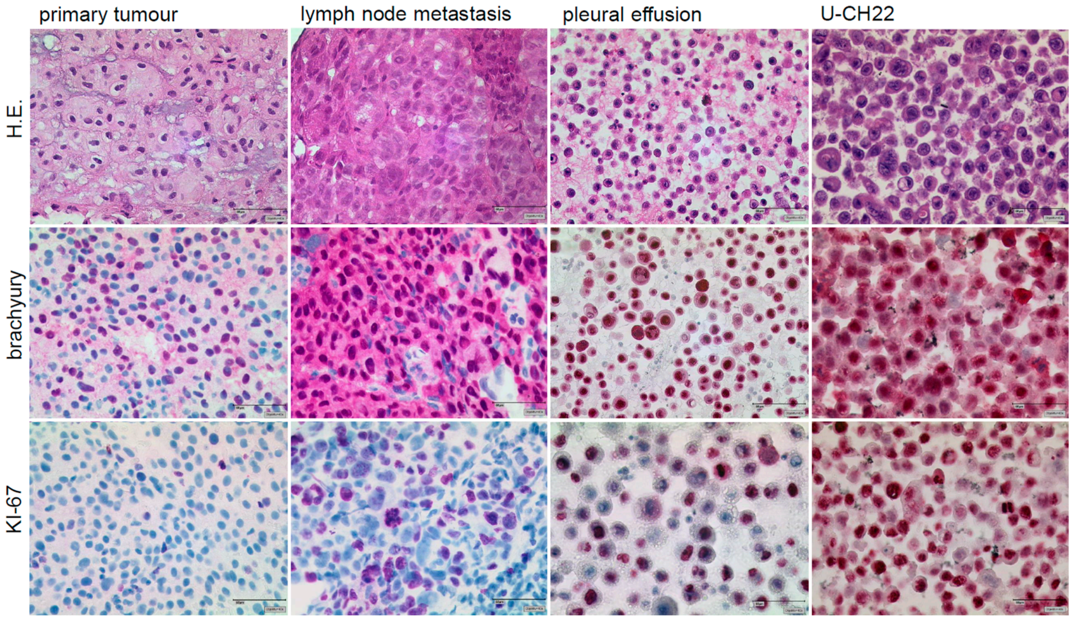

Figure S4. Comparison of proliferation rates of the primary chordoma and the ultimate metastases. The H/E staining and immunohistology/cytology for brachyury and KI-67 are given for the primary tumour, a lymph node metastasis, the cells isolated from a pleural effusion and the cell line U-CH22.

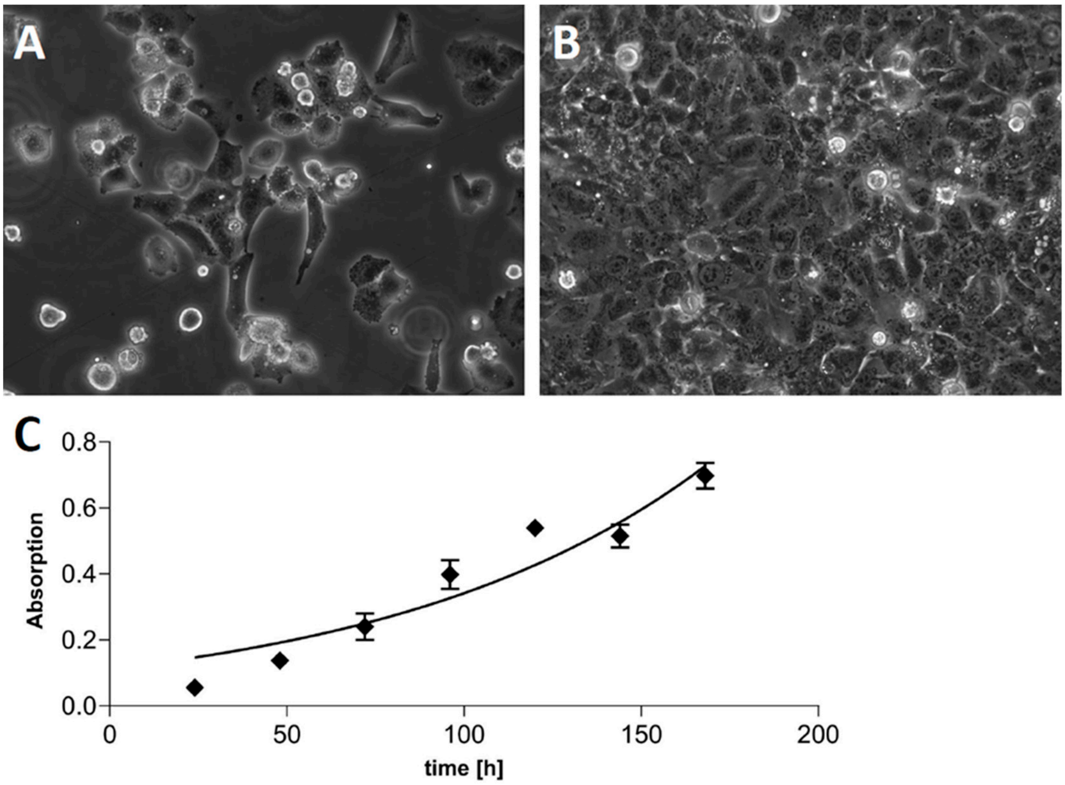

Figure S5. Phase contrast picture of cultured U-CH22 cells at about 50% confluence (A) and 100% confluence (B); growth curve of U-CH22; the x-axis represents the time and the y-axis the measured absorption parallel to the cell count (C).

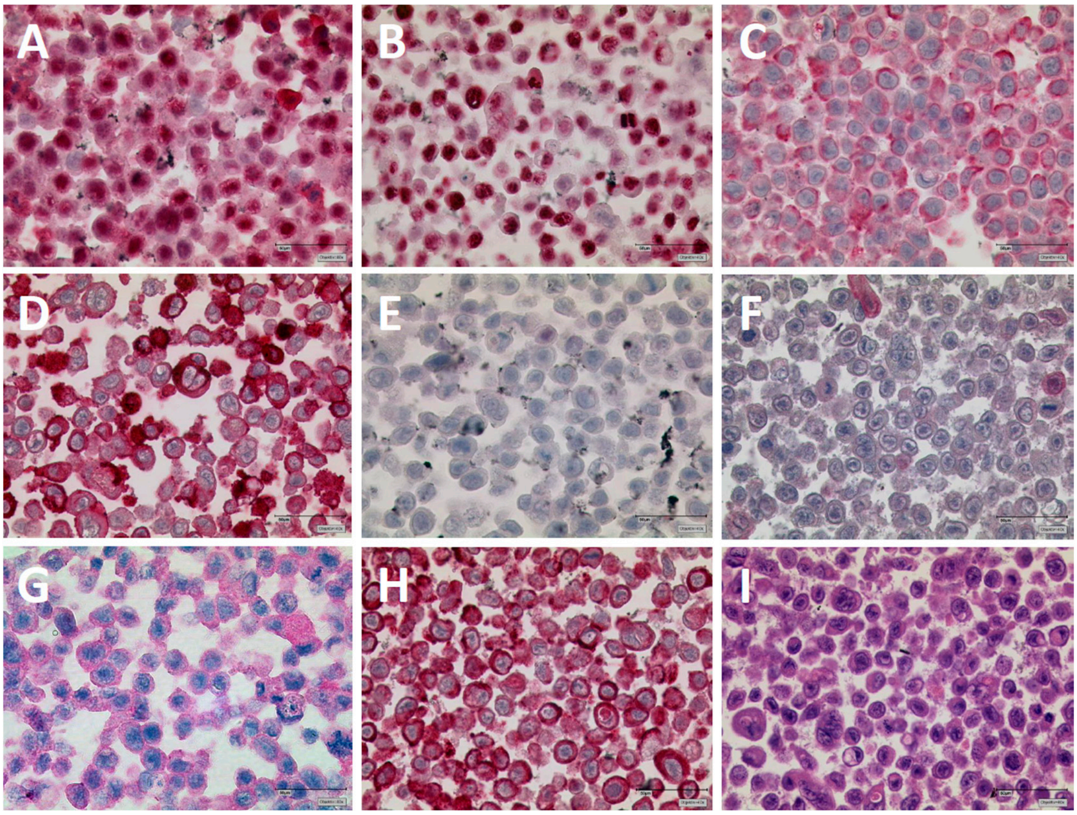

Figure S6. Immunocytochemistry of U-CH22 at 40x magnification; the following (immunocytochemical) stainings are shown: (A) brachyury, (B) KI-67, (C) vimentin, (D) EMA, (E) p53, (F) S100, (G) INI-1, (H) pan-cytokeratin, and (I) H/E.

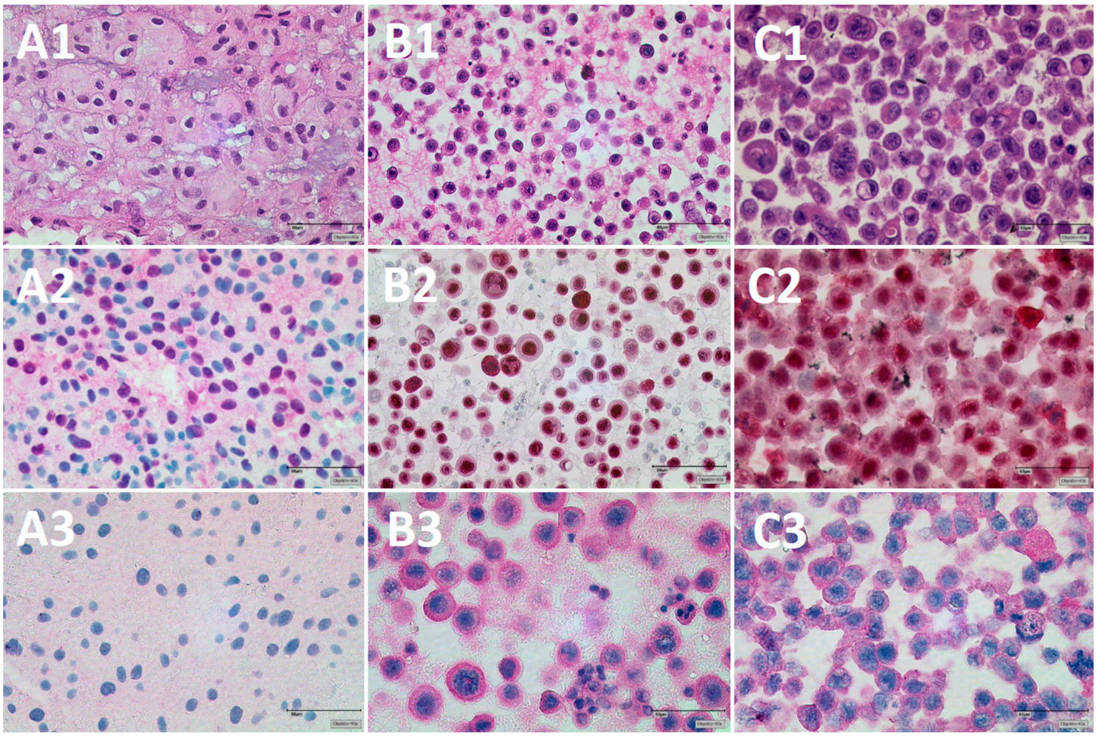

Figure S7. Morphology and immunohistochemistry: primary chordoma (A), pleural effusion, (B) and U-CH22 cell line (C); the following antibody

stainings are shown: H/E (A1-C1), brachyury (A2-C2), and INI-1 (nuclear staining; A3-C3).

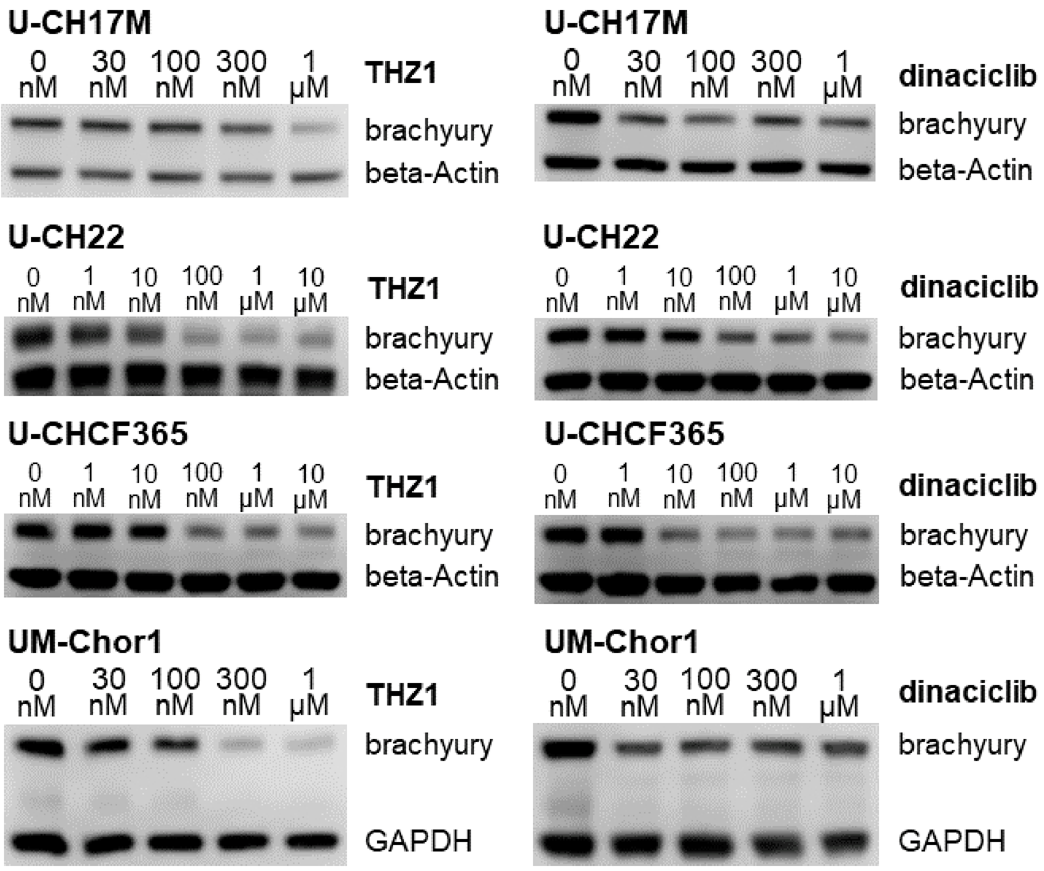

Figure S8. Exemplary Western Blots used to generate Figure 4; inhibitors THZ1 and dinaciclib were incubated in concentrations between 1 nM and 10 µM with 9 different chordoma cell lines for 24 hours (pictured here are U-CH17M, U-CH22, U-CHCF365, and UM-Chor1), GAPDH and beta-Actin were used as loading control proteins.

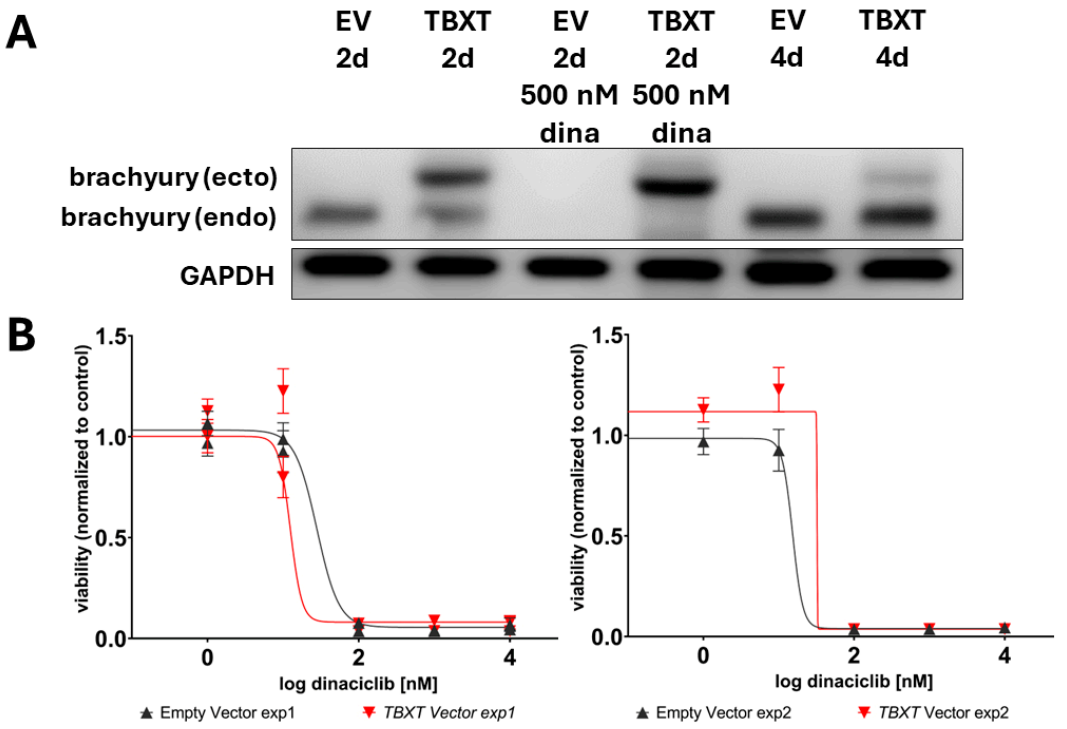

Figure S9. Effects of TBXT overexpression in dinaciclib-treated U-CH22 cells. A) Western blot detection of brachyury levels in U-CH22 cells transfected with either an empty vector (EV) or a TBXT-ORF-myc vector (TBXT), incubated with or without 500 nM of dinaciclib. Endogenous brachyury levels were diminished after dinaciclib treatment and ectopic brachyury was unaffected. Expression of ectopic brachyury persisted over the course of 4 days but was clearly reduced. B) Two replicates of viability measurements of U-CH22 cells treated with rising concentrations of dinaciclib. IC50 values of U-CH22 cells transfected with an empty vector control (black) or a TBXT-ORF-myc vector (red) averaged 21.6 nM and 22.4 nM, respectively. .
